# MRI analysis of distal tibiofibular joint and ankle anatomy to assess lateral ankle sprain risk

**DOI:** 10.1186/s13244-025-02102-6

**Published:** 2025-10-04

**Authors:** João Vieira, Ana Catarina Vieira, Alberto Vieira

**Affiliations:** 1 Radiology Department, Hospital Divino Espírito Santo, Ponta Delgada, Portugal; 2 Radiology Department, Hospital CUF Porto, Porto, Portugal; 3 Faculty of Medicine — University of Porto, Porto, Portugal

**Keywords:** Magnetic resonance imaging, Ankle injuries, Ankle joint

## Abstract

**Objective:**

To evaluate the risk of lateral ankle sprain (LAS) related to bone anatomical variations of the distal tibiofibular syndesmosis (DTS) and the height of both malleolar articular surfaces.

**Materials and methods:**

This retrospective cohort study included patients undergoing evaluation and assessment of quantitative parameters of the DTS and height of both malleolar articular surfaces in ankle MRI. Of the 216 patients included, 116 suffered LAS (53.7%). The measurements of the DTS were: anterior facet length of the fibular notch (*a*), posterior facet length of the fibular notch (*b*), angle between the anterior and posterior facets (*c*), fibular notch depth (*d*), tibial thickness (*e*), and fibular thickness (*f*). A subjective morphological analysis of the DTS was also assessed. The measurements of the malleolar articular surface length were: medial malleolar articular surface height (*h*), lateral malleolar articular surface height (*i*), and width of the talar dome articular surface (*j*).

**Results:**

Evaluating the DTS, patients with LAS showed a greater *c* (*p* < 0.001), a higher *a*/*b* (*p* = 0.013), and a lower *d*/*e* (*p* < 0.001). A plane DTS was also found to be a risk factor for sprain. Additionally, patients with LAS exhibited a lower *i*/*j* (*p* = 0.003). Indeed, the values of *c*, *a*/*b*, and *i*/*j* were independent predictors of LAS (*p* < 0.001, *p* = 0.015, *p* = 0.011).

**Conclusion:**

Anatomical factors of the DTS and lateral malleolus articular surface were predictors of the presence of LAS, mainly the angle and ratio between the anterior and the posterior facets and the ratio between the lateral malleolar height and the width of the talar articular surface.

**Critical relevance statement:**

Lateral ankle sprains are one of the most common musculoskeletal injuries, and the predisposing anatomical factors are not clear. This study correlates anatomical variants of the distal tibiofibular syndesmosis and the height of both malleoli with lateral ankle sprain.

**Key Points:**

- What specific bony variations in the distal tibiofibular syndesmosis and malleolar-talus relationship may predispose individuals to lateral ankle sprains?
- A shallow fibular notch, flat-type syndesmosis, and a lower lateral malleolar height/talar width ratio are more often found in first lateral ankle sprains.
- Understanding these anatomical variations may aid in injury prevention and improve risk assessment.

**Graphical Abstract:**

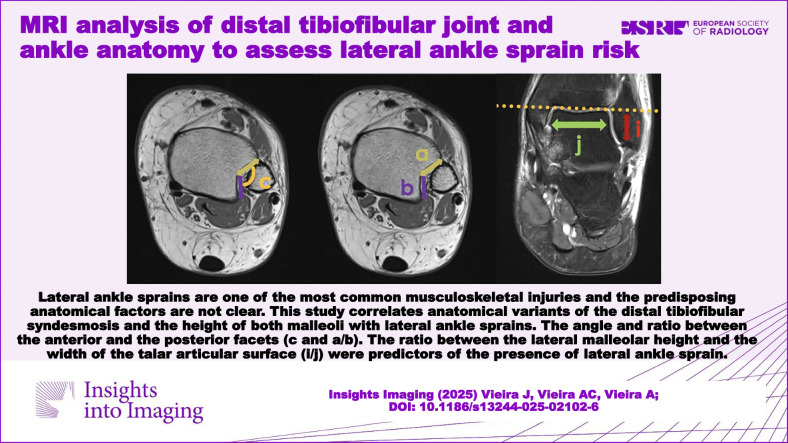

## Introduction

Ankle sprains are among the most prevalent musculoskeletal injuries, with a significant social and economic impact and in both athletes and the general population [1]. This pathology is responsible for up to 30% of all musculoskeletal injuries sustained by athletes and young people in the United States [2–4]. Notably, around 80% of ankle sprains affect the lateral aspect of the ankle [5, 6].

Given the high prevalence and potential for long-term morbidity, prevention of ankle sprains is a crucial focus. Effective prevention strategies require an understanding of intrinsic patient-specific risk factors. Recognised risk factors in athletes, particularly males, include a previous history of ankle sprain, higher body mass index, poor dynamic balance performance and reduced hip abduction strength [7]. Inadequate muscular strength around the ankle and hip joints has also been associated with an increased risk of ankle sprain [8, 9].

The complex anatomy of the ankle comprises three joints: the talocrural joint, the distal tibiofibular syndesmosis (DTS), and the subtalar joint. The ligamentous support of the talocrural joint comes from the joint capsule, the lateral ligament complex and the deltoid ligament [10].

The typical injury mechanism of lateral ankle sprain (LAS) is an inversion movement of the ankle, often accompanied by plantar flexion and excessive supination. This excessive inversion movement can lead to the injury of one of the lateral ankle ligaments, namely the anterior talofibular ligament (ATFL), the calcaneofibular ligament (CFL) and less commonly the posterior talofibular ligament (PTFL) [10].

Numerous studies have explored the aetiological and biomechanical factors contributing to ankle injuries [10–12]. However, there remains a gap in understanding the anatomical aspects, particularly in terms of bony constraint within the DTS and the talocrural joint, that may predispose individuals to ankle sprains.

**Fig. 1:**
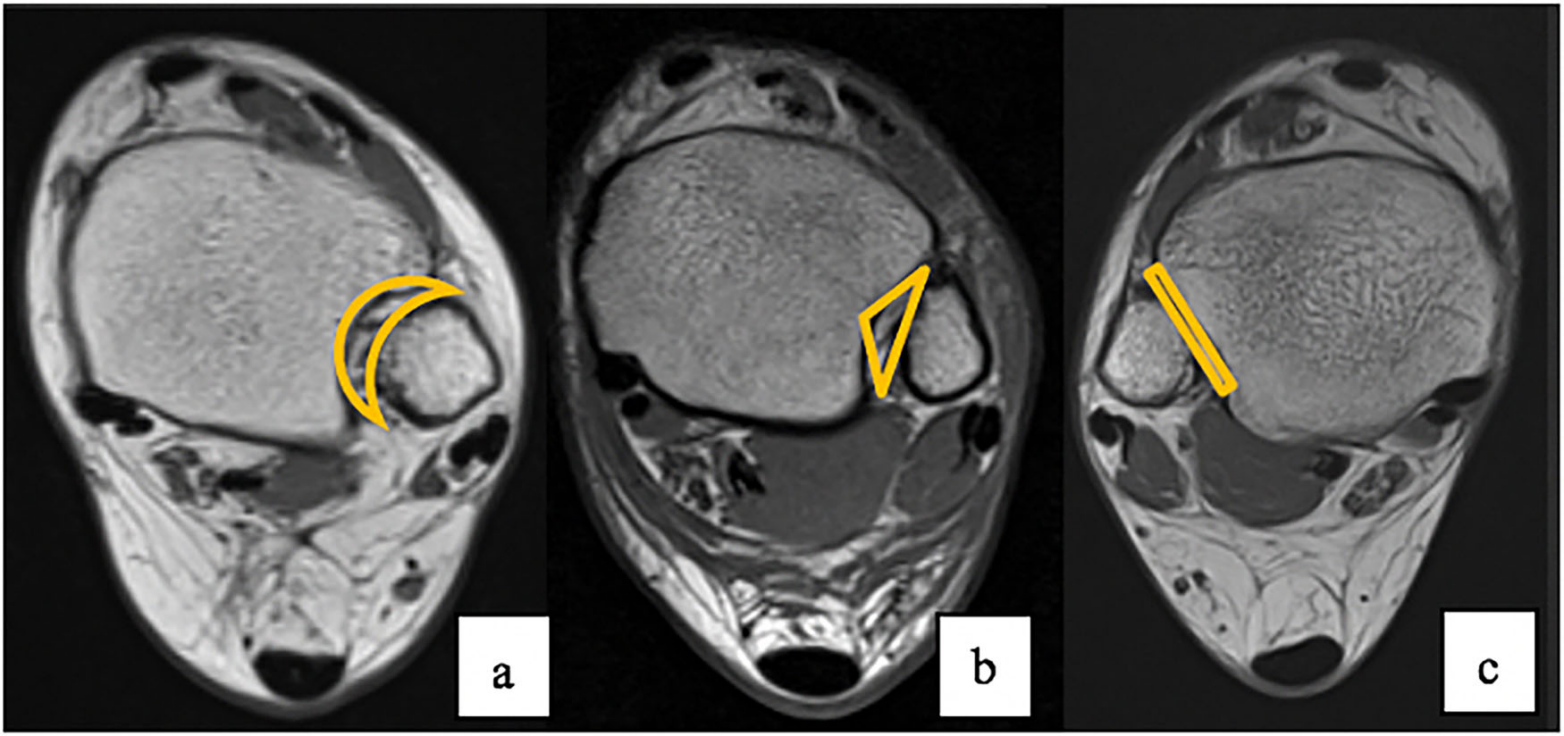
MRI T1-weighted images on the axial plane: Different morphologies of the distal tibiofibular joint anatomy. **a** Crescent-shaped; **b** Triangular-shaped; **c** Flat-shaped

The DTS is a joint characterised by micro-movements. Normal ankle function requires rotation, translation and inferior/superior movement of the fibula in the syndesmosis to accommodate the trapezoidal shape of the talus [13]. Previous research has shown that DTS bone structures exhibit considerable variability, both in subjective assessments of syndesmosis morphology and in parametric measurements of the intervenient bones [13–16] (Fig. 1). It was hypothesised that the DTS morphology has a role as an anatomical predisposing factor for LAS and that certain morphological characteristics may be more prevalent among individuals who have suffered LAS.

Another intrinsic structural factor that may contribute to LAS susceptibility is the degree of bony constraint within the talocrural joint, specifically the talus-malleolar relationship. Several studies have attempted to evaluate the risk of LAS by assessing malleolar height and talus dome width ratio using conventional radiography, but results have been inconclusive [17, 18]. It was hypothesised that the height of the malleolar articular surfaces, which can be readily assessed by magnetic resonance imaging (MRI), may be an anatomical predisposing factor to LAS.

The aim of this study was to assess the bony variations of the DTS and the height of both malleolar articular surfaces using MRI and to establish potential associations between these anatomical factors and the presence or absence of clinically and imaging (MRI-confirmed) recent LAS.

## Materials and methods

### Study design and patients

This is a single-centre, retrospective cohort study that was approved by the institutional review board at our hospital, Ethics number: 21/2024/48281570. Given the retrospective nature of the study, an exemption from participant consent was granted. The study was conducted in accordance with the World Medical Association Declaration of Helsinki.

**Fig. 2:**
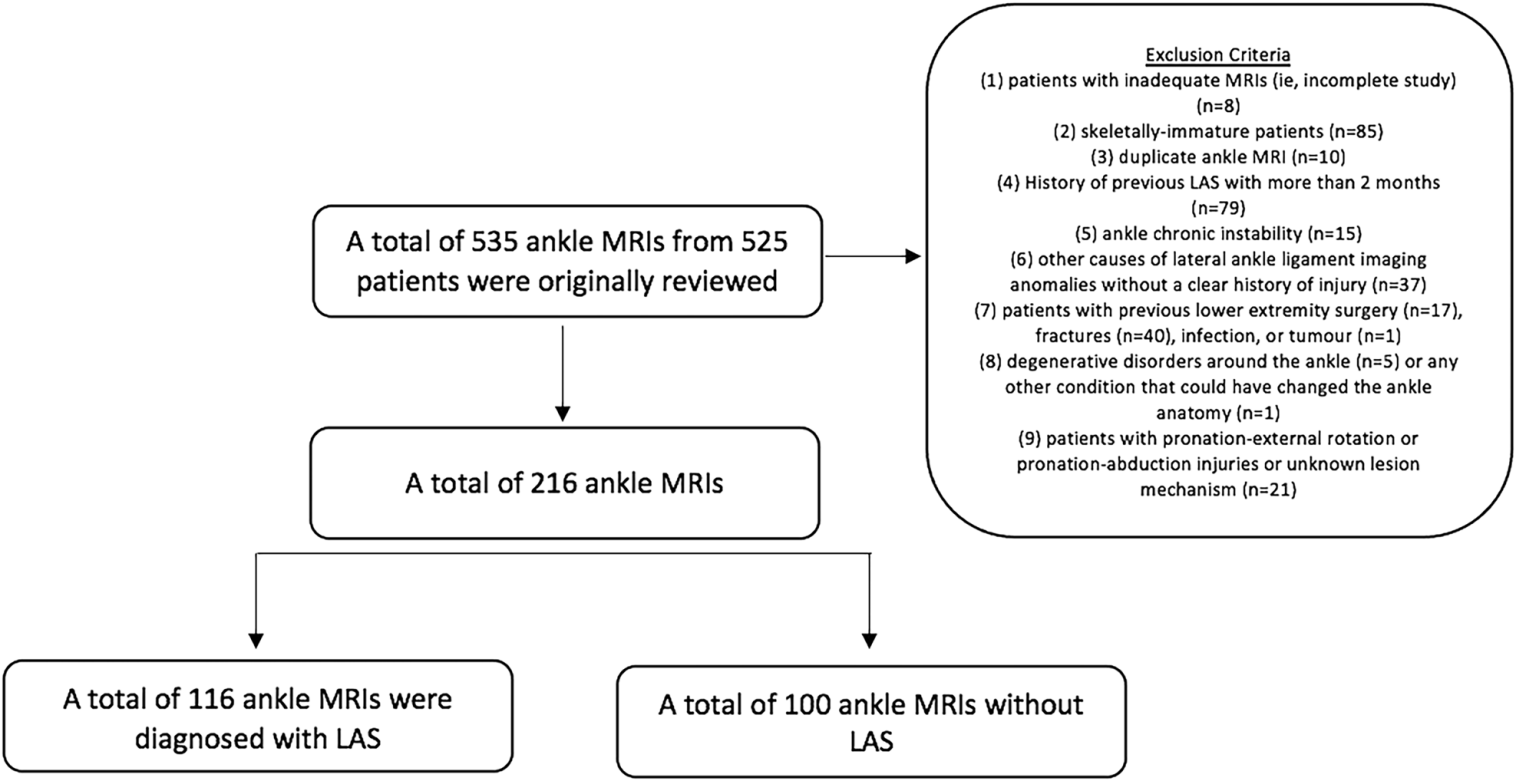
Illustration of excluded cases and exclusion criteria resulting in a final group of 116 ankle MRIs diagnosed with LAS, and another group with 100 ankle MRIs without LAS diagnosed

Patients who underwent ankle MRI between January and December 2023 were consecutively included in the study. A total of 535 ankle MRI studies from 525 patients were initially reviewed. The following exclusion criteria were applied: patients with inadequate MRI studies (*n* = 8); skeletally immature patients (*n *= 85); duplicate ankle MRIs from the same patient (*n* = 10); history of LAS with more than 8 weeks (*n* = 79); ankle chronic instability (*n* = 15); other causes of lateral ankle ligament imaging anomalies without a clear history of injury (*n *= 37); patients with previous lower extremity surgery (*n* = 17), fractures (*n* = 40), infection, or tumour (*n* = 1); degenerative disorders around the ankle (*n* = 5) or any other condition altering ankle anatomy (*n* = 1); patients with pronation-external rotation, pronation-abduction injuries or unknown lesion mechanism (*n *= 21) (Fig. 2).

Ultimately, 216 ankle MRIs from 216 patients were included in the study, constituting a convenience sample. Clinical data was collected from electronic medical records and included patient age, sex, and the affected ankle side.

### MRI evaluation

The MRI scans were performed using either a 3.0-T MRI scanner (MAGNETON Vida; Siemens) or a 1.5-T MRI scanner (MAGNETON Aera; Siemens), both utilising a similar dedicated 16-channel phased array coil for high-resolution imaging of the foot and ankle. The ankle was positioned in a neutral position, with the foot making a 90° angle with the ankle to avoid plantar or dorsiflexion. The MRI protocol included an axial T1-weighted sequence (3.0-T MRI scanner—repetition time (TR) 627 ms, echo time (TE) 12 ms, matrix 411 × 160, field of view (FOV) 160 mm, and slice thickness 2.5 mm; 1.5-T MRI scanner—TR 593 ms, TE 11 ms, matrix 437 × 170, FOV 160 mm, and slice thickness 2.5 mm) and a coronal intermediate fat-suppression weighted sequence (3.0-T MRI scanner—TR 3130 ms, TE 44 ms, matrix 463 × 180, FOV 160 mm, and slice thickness 2.5 mm; 1.5-T MRI scanner—R 5400 ms, TE 32 ms, matrix 488 × 190, FOV 160 mm, and slice thickness 2.5 mm), both sequences were the primary ones employed for MRI measurements. In addition, a sagittal and coronal T1-weighted sequence, as well as a sagittal STIR and an axial proton-density fat-saturated sequence, were acquired for diagnostic purposes (interpretation of LAS and concomitant injuries).

### MRI interpretation

LAS diagnosis was established based on a documented history of the first-time ankle inversion injury occurring within the previous eight weeks, combined with MRI evidence of a sprain in the ATFL, CFL, and/or PTFL. In the context of MRI evaluation, the diagnosis of LAS is determined by meticulous evaluation of the ATFL, the CFL, and the PTFL. Ligament injuries were classified as unremarkable, sprained (high signal intensity without structural ligament discontinuity), partially torn (partial structural discontinuity), or completely torn (total structural discontinuity) [19].

Two experienced musculoskeletal radiologists (with 7 and 33 years of experience) diagnosed LAS in 116 patients. Concomitant injuries, including bone marrow oedema around the articular surfaces, acute osteochondral lesions of the talar dome, and deltoid ligament injuries, were also assessed.

An acute osteochondral lesion was defined as areas of diffuse hyperintensity of the talar dome, directly adjacent to the subchondral plate, with or without cartilage surface damage [20]. In the inversion mechanism of injury, the medial collateral (deltoid) ligament complex can be injured due to tibio-talar compression, resulting in oedema and/or ligament tears [19]. The presence of pathology in the medial ligamentous complex was also assessed in each patient.

**Fig. 3:**
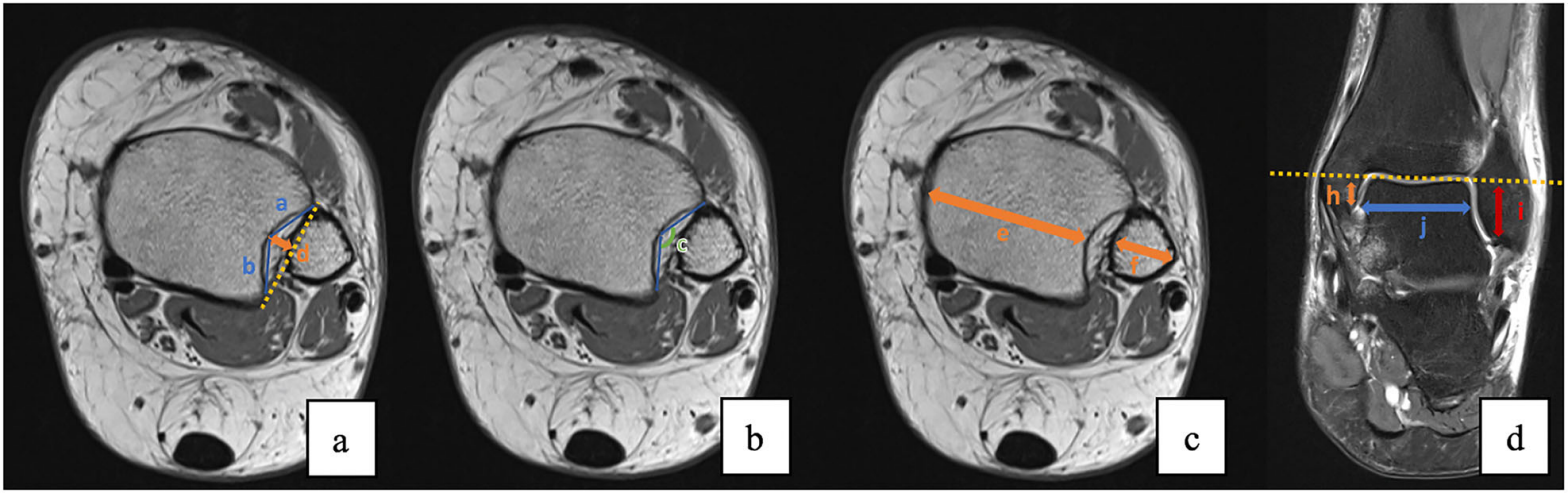
MRI T1-weighted images on the axial plane: **a**–**c**. MRI T2-weighted image on the coronal plane **d**. Representation of the measurements made on MRI sections

In all 216 patients, the DTS morphology and parametric measurements were assessed using an axial T1-weighted sequence, with measurements performed 1 cm above the tibio-talar joint. The following parameters were measured: the anterior facet length of the fibular notch (*a*), the posterior facet length of the fibular notch (*b*), the angle between the anterior and the posterior facets (*c*), the fibular notch depth (*d*), the tibial thickness (*e*), and the fibular thickness (*f*) (Fig. 3).

A subjective morphological classification of the DTS was also performed, categorising it into crescent-shaped, triangular-shaped, or flat-shaped configurations (Fig. 1).

Additionally, several ratios were calculated to analyse anatomical relationships: the anterior to the posterior facet length (*a*/*b*); the fibular notch depth to the tibial and to the fibular thickness (*c*/*e* and *c*/*f*, respectively); the tibial to the fibular width (*e*/*f*); and the sum of the anterior and posterior facets length relative to the tibial thickness ((*a* + *b*)/*e*).

The degree of bony constraint within the talocrural joint, specifically the talus-malleolar relationship, was assessed on coronal intermediate fat-suppression weighted sequences, with measurements performed at the plane of maximum lateral malleolar articular surface length. The parameters measured were the medial malleolar articular surface height (*h*), the lateral malleolar articular surface height (*i*), and the talar articular surface width (*j*) (Fig. 3).

Also, for the talocrural joint, several ratios were calculated to analyse anatomical relationships: the medial malleolar articular surface height to the talar articular surface width (*h*/*j*), the lateral malleolar articular surface height to the talar articular surface width (*i*/*j*), and the lateral malleolar articular surface height to the medial malleolar articular surface height (*i*/*h*).

All MRI measurements, including both quantitative (parametric) and qualitative (morphological) analyses, were performed by a musculoskeletal fellow with one year of experience, who was blinded to the final MRI diagnosis to minimise bias.

### Statistical analysis

Categorical variables were reported as frequencies and percentages, while continuous variables were presented as means and standard deviations (SD) for normally distributed data, or as medians and interquartile range (IQR) otherwise. The tests used to assess the normality of the distribution of the variables were the Kolmogorov-Smirnov test and the Shapiro-Wilk test. Comparisons between categorical variables were performed using the Chi-square or Fisher’s exact test. Means and medians of continuous variables were compared using an independent group *t*-test or Mann–Whitney *U*-test, respectively. Multivariable analysis was performed using a binary logistic regression including statistically significant variables on univariable analysis and excluding those with multicollinearity, to identify independent predictive factors associated with LAS.

A *p*-value less than 0.05 was considered statistically significant. Statistical analysis software IBM SPSS version 29.0 (IBM Corp.) was used for all tests performed.

## Results

A total of 216 ankle MRIs from 216 patients were included in the study. Among the 116 patients diagnosed with LAS, the mean age was 39 ± 13 years, with 68 males (59%) and 48 females (41%). In the 100 patients without LAS, the mean age was 43 ± 13 years, with 46 males (46%) and 54 females (54%). There were no statistically significant differences regarding gender, age and affected ankle side between those with and without LAS (*p* = 0.064, *p* = 0.054 and *p* = 0.459, respectively). The demographic characteristics of both groups are detailed in Table 1.

**Table 1:** Patient demographics in the groups with and without lateral ankle sprain

	LAS group (*n* = 116)	Without LAS group (*n* = 100)	*p*
Sex: *n* (%)			0.064
•Female	48 (41.4)	54 (54)	
•Male	68 (58.6)	46 (46)	
Age, in years: mean ± SD	39 ± 13	43 ± 13	0.054
Ankle: *n* (%)			0.459
•Right	65 (56)	51 (51)	
•Left	51 (44)	49 (49)	

*LAS* lateral ankle sprain, *SD* standard deviation

In the cohort of patients diagnosed with LAS, the most frequently affected ligament was the ATFL in 114 patients (98%), followed by the CFL in 102 patients (88%) and the PTFL in 8 patients (7%).

The pattern of lateral ligament injuries and concomitant injuries in the LAS group is detailed in Table 2.

**Table 2:** Ligamentous lesions and Concomitant Injuries in the lateral ankle sprain group

	Ligamentous lesions and concomitant injuries (*n* = 116)
ATFL: *n* (%)	
•Completely Torn	30 (26)
•Partially Torn	42 (36)
•Sprain	42 (36)
•Normal	2 (2)
CFL: *n* (%)	
•Completely Torn	1 (1)
•Partially Torn	33 (28)
•Sprain	68 (59)
•Normal	14 (12)
PTFL: *n* (%)	
•Sprain	8 (7)
•Normal	108 (93)
Deltoid injury: *n* (%)	
•Present	74 (64)
•None	42 (36)
Concomitant bone injury: *n* (%)	
•Osteochondral Lesion	5 (4)
•Bone marrow oedema	54 (47)
•None	57 (49)

*ATFL* anterior talofibular ligament, *CFL* calcaneofibular ligament, *PTFL* posterior talofibular ligament

The results summarised in Table 3 compare DTS characteristics on axial T1-weighted sequence between LAS patients and controls.

**Table 3:** Comparison of the various distal tibiofibular syndesmosis characteristics between individuals with and without lateral ankle sprain

	LAS group (*n* = 116)	Without LAS group (*n* = 100)	*p*
*a*, in mm: median (IQR)	12.6 (3.6)	11.8 (2.7)	**0.011**
*b*, in mm: mean ± SD	12 ± 2.5	12.6 ± 2.3	0.060
*c*, in degrees: mean ± SD	135 ± 9°	130 ± 9°	**< 0.001 **
*d*, in mm: mean ± SD	4.4 ± 1.2	5.0 ± 1.2	**< 0.001**
*e*, in mm: mean ± SD	40.6 ± 3.4	39.6 ± 3.7	**0.034**
*f*, in mm: mean ± SD	14.4 ± 1.8	14.5 ± 1.6	0.430
*a*/*b*: median (IQR)	1.02 (0.5)	0.94 (0.2)	**0.013**
*d*/*e*: mean ± SD	0.11 ± 0.03	0.13 ± 0.03	**< 0.001**
*d*/*f*: mean ± SD	0.31 ± 0.07	0.34 ± 0.08	**0.001**
*e*/*f*: mean ± SD	2.86 ± 0.33	2.75 ± 0.33	**0.013**
(*a* + *b*)/*e*: mean ± SD	0.61 ± 0.06	0.62 ± 0.06	0.430
Distal ankle syndesmosis types: *n* (%)			
•Flat	29 (25)	11 (11)	**0.008**
•Triangular or “V” shape	49 (42.2)	43 (43)	0.910
•Crescent	38(32.8)	46 (46)	**0.047**

*LAS* lateral ankle sprain, *SD* standard deviation, *IQR* interquartile range, *a* anterior facet length of the fibular notch, *b* posterior facet length of the fibular notch, *c* angle between the anterior and posterior facets, *d* fibular notch depth, *e* tibial thickness, *f* fibular thickness

Statistically significant *p*-values and odds ratios are highlighted in bold

The anterior facet length of the fibular notch (*a*) was significantly greater in the LAS group 12.6 (3.6) vs. 11.8 (2.7) mm, *p* = 0.011). The angle between the anterior and posterior facets of the fibular notch (*c*) was significantly larger in the LAS patients (135 ± 9° vs. 130 ± 9°, *p* < 0.001). Furthermore, the tibial thickness (*e*) was greater in individuals with LAS (40.6 ± 3.4 vs. 39.6 ± 3.7 mm, *p* = 0.034). Conversely, the fibular notch depth (*d*) was significantly lower in the LAS group (4.4 ± 1.2 vs. 5.0 ± 1.2 mm, *p* < 0.001).

The ratio analysis further revealed that *a*/*b* ratio was significantly higher in the LAS group (1.02 (0.5) vs. 0.94 (0.2), *p* = 0.013). Both *d*/*e* and *d*/*f* ratios were significantly lower in the LAS group compared with the controls (0.11 ± 0.03 vs. 0.13 ± 0.03, *p* < 0.001 and 0.31 ± 0.07 vs. 0.34 ± 0.08, *p* = 0.001, respectively). However, the ratio (*a* + *b*)/*e* did not show a statistically significant difference (0.61 ± 0.06 vs. 0.62 ± 0.06, *p* = 0.430).

The subjective morphological analysis of the DTS has shown that the flat-type DTS was significantly more prevalent in the LAS group (*p* = 0.008), whereas the crescent-type DTS was less frequent (*p* = 0.047).

The heights of the lateral and medial malleolar articular surfaces were compared between the groups in Table 4.

**Table 4:** Comparison of the height of the lateral and medial malleolar articular surfaces and the width of the talar articular surface between individuals with and without lateral ankle sprain

	LAS group (*n* = 116)	Without LAS group (*n* = 100)	*p*
*h*, in mm: mean ± SD	12.3 ± 2.0	12.4 ± 1.7	0.464
*i*, in mm: mean ± SD	21.2 ± 3.0	21.9 ± 2.6	0.054
*j*, in mm: mean ± SD	37.0 ± 3.0	36.5 ± 3.0	0.221
*h*/*j*: mean ± SD	0.33 ± 0.05	0.34 ± 0.04	0.111
*i*/*j*: mean ± SD	0.57 ± 0.07	0.60 ± 0.07	**0.003**
*i*/*h*: mean ± SD	1.76 ± 0.29	1.79 ± 0.29	0.401

*LAS* lateral ankle sprain, *SD* standard deviation, *h* medial malleolar articular surface height, *i* lateral malleolar articular surface height, *j* width of the talar dome articular surface

Statistically significant *p*-values and odds ratios are highlighted in bold

The *i*/*j* ratio was significantly lower in the LAS group (0.57 ± 0.07 vs. 0.60 ± 0.07, *p* = 0.003), whereas other ratios assessed, including *h*/*j* (0.33 ± 0.05 vs. 0.34 ± 0.04, *p* = 0.111) and *i*/*h* (1.76 ± 0.29 vs. 1.79 ± 0.29, *p* = 0.401), did not show statistically significant differences.

A logistic regression analysis was conducted in order to ascertain significant independent predictors of ankle sprain. The analysis identified three variables: the angle between the anterior and posterior facets of the fibular notch (*c*) (*p* < 0.001), the *a*/*b* ratio (*p* = 0.015) and the *i*/*j* ratio (*p* = 0.011). These results are summarised in Table 5.

**Table 5:** Logistic regression analysis identifies three variables as significant independent predictors

	*B*	Exp (*B*)	Confidence interval	*p*
*c*	0.055	0.017	1.023–1.091	**< 0.001**
*a*/*b*	1.012	2.750	1.218–6.21	**0.015**
*i*/*j*	−5.750	0.003	0.000–0.266	**0.011**

*c* angle between the anterior and posterior facets, *a* anterior facet length of the fibular notch, *b* posterior facet length of the fibular notch, *i* lateral malleolar articular surface height, *j* width of the talar dome articular surface

Statistically significant *p*-values and odds ratios are highlighted in bold

## Discussion

The lateral collateral ligament complex, composed of the ATFL, CFL, and PTFL, acts as a single functional unit [21].

The ATFL is the most frequently injured ligament in ankle sprains, typically due to a plantar flexion-inversion mechanism. Isolated CFL injuries are rare and usually occur with ATFL injuries, while the PTFL is rarely affected in low-grade sprains [19, 22, 23].

Our findings align with the literature; the ATFL was the most frequently injured ligament, followed by the CFL and the PTFL, with only two patients exhibiting isolated CFL injuries. While previous studies report that only up to 20% of ATFL lesions are associated with CFL injuries [22, 24], it is important to highlight that, in the LAS group, the primary indications for undergoing an ankle MRI were unsatisfactory clinical progression (as persistent pain and swelling), suspected concomitant injuries or concern for severe ligamentous injury. Consequently, the study cohort likely represents cases with higher sprain severity, which may explain the increased prevalence of concomitant ATFL and CFL injuries (86%), as well as the high prevalence of deltoid ligament injuries and bone injuries.

Several studies have indicated that the anatomical bone variations of the ankle joint influence the outcome of an inversion injuries, either leading to a fracture or ligament damage [17, 25]. The ankle’s anatomical bone variations can be observed on both the DTS and the talocrural joint [13, 16, 17]. To the best of our knowledge, this study is the first to recognise in patients with the first episode of ankle sprain the presence of certain bony variations of the DTS (analysed both quantitatively and qualitatively), and the talocrural joint can be more prevalent in a population with recent LAS.

**Fig. 4:**
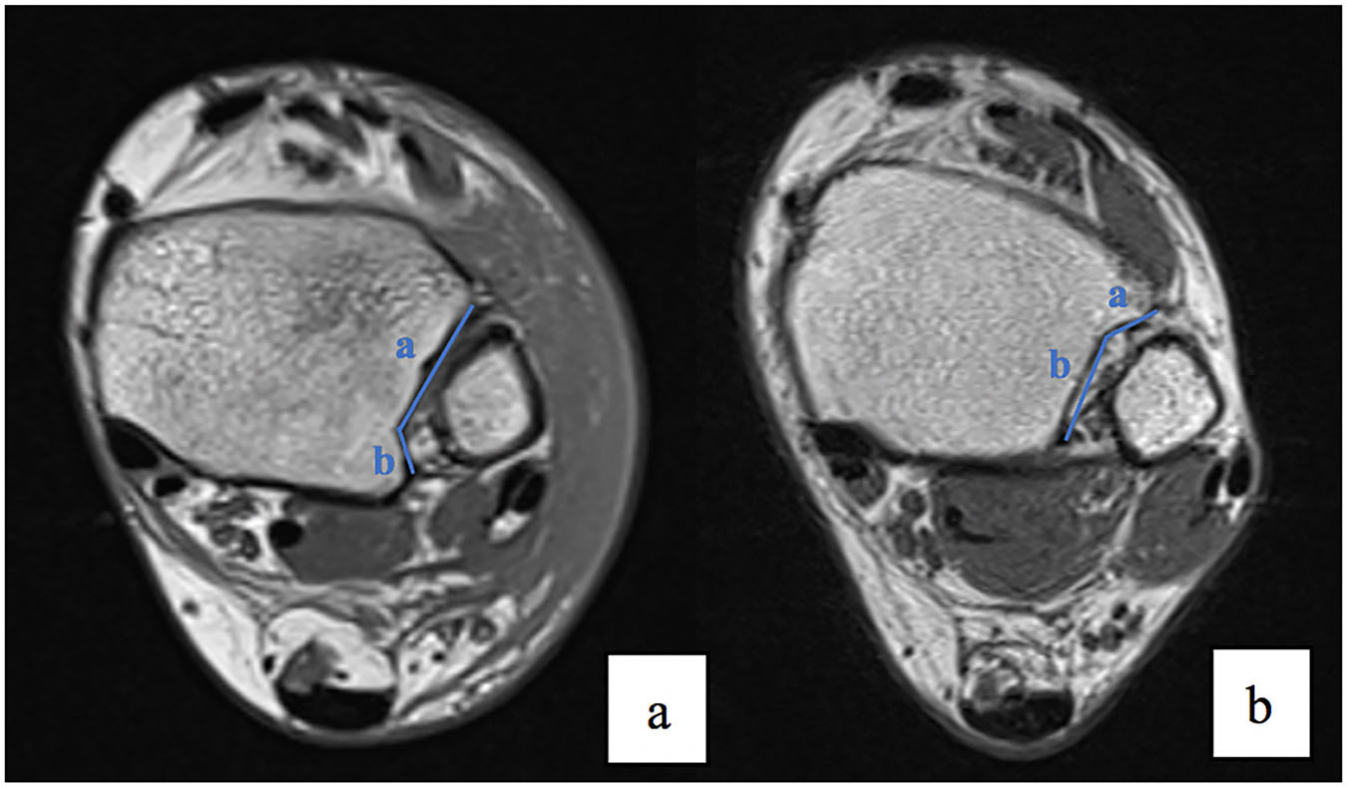
MRI T1-weighted images on the axial plane, at the same level, 1 cm above the tibio-talar joint of two different patients. Note the different morphology of the DTS. **a** A patient with a posterior position of the maximum measurable depth of the fibular notch, with a calculated *a*/*b* ratio of 2.43. **b** A patient with a lower *a*/*b* ratio of 0.61, with a smaller anterior facet length of the fibular notch when compared with the posterior facet length, due to a more prominent anterior tibial tubercle

The DTS variations observed in the LAS group, including the increased anterior facet length of the fibular notch (*a*), the greater tibial thickness (*e*), the decreased fibular notch depth (*d*), and a higher anterior-posterior facet angle (*c*), suggest that a shallower tibiofibular notch and an elongated anterior facet may predispose individuals to ATFL injuries. Furthermore, the study revealed that the *a*/*b* and *e*/*f* ratios were significantly higher in the LAS group, while the *d*/*e* and *d*/*f* ratios were significantly lower when compared with the control group. Our explanation is that the more posterior the maximum measurable depth of the fibular notch, the higher the measure of the anterior facet length of the fibular notch, and both of these factors may contribute to a lower anterior fibular constraint (Fig. 4).

A previous study, which evaluated the bony DTS measurements in a smaller cohort of patients with proven ankle instability, found an inferior anterior facet length in the study group [15]. Our study emphasises a different population with a recent proven LAS diagnosis, without focusing on the recurrent ankle sprain outcome. Also, the higher *a*/*b* ratio found in the LAS group strengthens our results. In our observation, a smaller anterior facet length of the fibular notch was associated with a more prominent anterior tibial tubercle, which prevents the forward slipping of the distal fibula. This observation is also supported by anatomical DTS studies [16].

To support the conclusions of the quantitative measurements, a morphological analysis of the DTS was also conducted. The shapes of the joint were classified into three distinct types based on the morphology of the incisura fibularis, a flat-shape was found to be more prevalent on the LAS group, in contrast with the crescent-shape more prevalent on the control group. Prior research on DTS morphology also found that a flat-shaped DTS is associated with higher ATFL injury risk and recurrent sprains [13].

On the assessment of the talocrural joint, the *i*/*j* ratio, which reflects the height of the lateral malleolar articular surface relative to talar dome width, was significantly lower in LAS patients, suggesting a possible anatomical relationship predisposing to sprains. In previous studies, no significant difference was found between malleolar height assessed by radiograph and the presence of ankle sprain [18]. To our knowledge, this is the first study comparing the height of the lateral malleolar articular surface and talar dome width on MRI in patients with and without LAS.

When the quantitative metrics were all analysed three independent factors were found to be statistically significant, two from the DTS, the *a*/*b* ratio and *c*, as well as the *i*/*j* ratio.

These findings suggested different risk factors may act independently in addition to each other to result in LAS in the study population.

Patients with anatomically unstable ankle configurations may be more prone to lateral sprain injuries, and these may be assessed in any ankle MRI study. Preventive strategies, especially in athletes, may be implemented when these study risk factors are found. A regular proprioceptive training and neuromuscular control exercises have been shown to reduce sprains and compensate for mechanical instability [26, 27].

The strengths of this study include: First, the use of MRI to clearly provide precise measurements of anatomical variation, the DTS joint and the talocrural joint in the same study. Second, the first study provides quantitative and qualitative information concerning the DTS and finds a correlation between them. Third, unlike previous studies, the LAS group consisted of patients with both clinically and radiologically confirmed recent LAS at the time of examination [17].

The main limitations of our study are its retrospective design, being a single-centre study and that two different magnets (1.5 T and 3 T) were used. Additionally, most ankles analysed in the control group presented some form of pathology and were therefore not entirely normal. Ideally, the control group would have consisted of MR images of completely healthy ankles. However, we carefully reviewed the medical history and MRI findings of the control group to exclude individuals with a prior history or current imaging evidence of ligamentous pathology. Another limitation of this study is the lack of analysis of other factors known to be risk factors of ankle sprain, such as higher body mass index, poor dynamic balance performance and reduced hip abduction strength [7]. Future studies incorporating these variables, along with other anatomical features, particularly of the talocrural joint and subtalar joint, are necessary to provide a more comprehensive understanding of LAS.

## Conclusion

LAS is a common injury, and a clear understanding of the relevant bony structures and the functional anatomy of the ankle complex, including the talocrural joint and the DTS, is essential to further identify anatomical structural predisposition. This study highlights the prevalence of specific bony variations in individuals with recent LAS and their potential role in injury predisposition. By providing new insights into the anatomical factors associated with LAS, these findings contribute to future multifactorial studies.

## Data Availability

Data supporting this study cannot be made available due to ethical and legal restrictions.

## Abbreviations

ATFL: Anterior talofibular ligament
CFL: Calcaneofibular ligament
DTS: Distal tibiofibular syndesmosis
FOV: Field of view
IQR: Interquartile range
LAS: Lateral ankle sprain
PTFL: Posterior talofibular ligament
SD: Standard deviation
TE: Echo time
TR: Repetition time
